# Supplementary material for: Effects of dual-task training on cognitive-motor learning and cortical activation: A non-randomized clinical trial in healthy young adults
Source: PLoS One. 2025 May 8;20(5):e0322036. doi: 10.1371/journal.pone.0322036 (PMC12061167; doi:10.1371/journal.pone.0322036)
Supplement: S2 Appendix — (DOCX) [file pone.0322036.s002.docx]

DT = Dual-task

RT = Reaction Time

fNIRS = Functional Near Infrared Spectroscopy

PFC = Prefrontal Cortex

HbO = Oxygenated Hemoglobin

Hb = Deoxygenated hemoglobin

SRT = Simple Reaction Time

CRT = Complex Reaction Time
