# Supplementary material for: Effects of dual-task training on cognitive-motor learning and cortical activation: A non-randomized clinical trial in healthy young adults
Source: PLoS One. 2025 May 8;20(5):e0322036. doi: 10.1371/journal.pone.0322036 (PMC12061167; doi:10.1371/journal.pone.0322036)
Supplement: S4 Protocol — (PDF) [file pone.0322036.s004.pdf]

Effects of Dual-Task Training on Cognitive and Motor Learning and Cortical Activation in Healthy Young Adults	
**Study Objectives**	Specific study objectives include: 1) assessment of the effects of dual-task training using a dynamic balance task and an auditory reaction time task on dual-task performance in healthy young adults and 2) to assess the cortical activity within the prefrontal and sensorimotor areas in response to dual-task training using fNIRS.
**Background & Rationale**	A dual-task is defined as simultaneous performance of motor-cognitive tasks, which represent a very large part of day-to-day activities. An example of a dual-task is walking while taking on the phone, where walking represents a motor task and talking is a cognitive task. The performance of one or both tasks deteriorates while performing the two tasks concurrently, which is termed dual-task interference. Concurrent execution of these tasks imposes demand on attentional capacity and resources. Altogether, since attention needs to be divided between both of the tasks, there is limited central processing capacity that results in dual-task interference. Dual-task interference affects functional performance.<br><br>Research of young adults in a dual-task situation has indicated significant performance decrements relative to situations that involve a motor or cognitive task only (Beauchet et al, 2005). In this cohort, dual-task training has resulted in significant improvements in cognitive and motor task performance under dual-task situations, suggesting efficiency of the intervention strategy (Kiss, Brueckner, Muehlbauer, 2018). However, investigation of the effects of dual-task practice on balance and cognitive task performance among healthy young adults has shown conflicting evidence (Ghai, Ghai, Effenberg, 2017). Improvements in both tasks versus improvements in the motor task only have both been reported in this cohort. Moreover, the dual tasks included in these studies are less complex in nature. Since postural control involves complex interactions of somatosensory, vestibular, and visual systems, it is important to challenge these systems in dual-task paradigm so that the effects could be directly translated to real life situations. Thus, further research is needed to clarify the impact of complex dual-task training on both balance and cognitive task performance in healthy young adults. Moreover, it is important to assess the retention of learning effects of such complex task, which would help in determining the dose of such interventions. In this prospective study, retention effects of dual-task training will be explored, and motor learning response will be mapped by functional near-infrared spectroscopy (fNIRS) assessment, which will benefit the existing literature of dual-task training that lacks this measure. Enhanced postural stability (especially under influence of dual-task requirements) should translate to preserved steadiness during everyday static and dynamic activities.<br><br>Dynamic postural stability is an integral aspect of postural control and it involves complex interaction of somatosensory, vestibular, and visual systems. However, a motor task involving complex interaction of these systems combined with cognitive task challenges has not been investigated thoroughly. Coordinated

	control of the body requires integration of all three systems (i.e. somatosensory, vestibular, and visual). It is important to systemically study the interference of an additional attention-demanding task, such as an auditory stimulus reaction time task on interaction of these systems that may contribute to decreased postural stability. Moreover, it is important to investigate if training on such a complex dual-task can reduce motor-cognitive interference and improve postural stability in complex dual-task conditions. The motor task (balance task) involving the dynamic stability platform (Lafayette Instrument, model 16030, Lafayette Instrument, Lafayette, IN) was chosen after extensive literature review of dual-task training. The balance task engages a wide range of brain systems, including vestibular, visual, motor, somatosensory, and cognitive, serving as a simple probe to determine the postural control and motor learning response to training. Enhancement of motor control and cognitive processing (learning) may be indicated by improved postural stability (i.e. balance performance) as well as cognitive task performance under dual-task condition, respectively. Participants will be instructed to stand on the stability platform with their feet facing forward. The goal of the task will be to keep the platform level for as long as possible during trails of 30 seconds (s). Performance for each trial will be determined by quantifying the cumulative amount of time that a participant will be able to maintain the platform within 3° of horizontal. Baseline performance and post-training testing will involve 18 trials each. Each participant will receive 5 days of training involving 18 trials/day. The cognitive task will involve an auditory reaction time task, which provides necessary complexity of performance requirements under dual-task condition.<br><br>Dynamic balance training in a dual-task setting has given rise to beneficial effects to postural stability in individuals with impaired balance capabilities. A dynamic stability platform task has previously been used in dual-task investigation of healthy young adults, and I seek to add insight to the existing literature involving this cohort and potentially highlight the importance of dual-task training in learning such a complex task. Specifically, an inverse relationship exists between dual-task complexity and postural stability. To lessen the likelihood of plateaus in motor and cognitive learning of the dual-task, this relationship is carefully considered in my investigation of healthy young adults (i.e. use of a complex auditory reaction time task).<br><br>I am trained and familiar with operation of the PsymLab Psychomotor Control Software as well as the StimSys software for measuring balance and reaction time, respectively, due to my undergraduate research experience in the ECU Pediatric Assessment and Rehabilitation Lab (PEARL).
**Study Design**	Single group repeated measures design.
**Groups**	Single group: Healthy young adults receiving dual-task training
**Number of Subjects & Power Analysis**	A power analysis was done based on our pilot dynamic stability platform data in healthy young adults. Using the standard deviation of change scores (2.5 s and 1.9 s in experimental and control groups respectively), a power of 0.80 and an alpha of

	0.05, 20 total subjects would be sufficient to detect a mean difference in change score of ≥ 3 s on our primary outcome measure.
**Inclusion & Exclusion Criteria**	**Inclusion Criteria:**<br>1. 18-40 years of age<br><br>**Exclusion Criteria:**<br>1. Cognitive deficits or communication problems<br>2. Impaired vision<br>3. Balance disorders such as vestibular disorders, etc.<br>4. Known cardiorespiratory dysfunctions<br>5. Presence of lower extremity condition, injury, or surgery within last 3 months which could compromise training
**Behavioral Training**	A combined motor-cognitive task will be used for behavioral training (i.e. dual-task training) on visits 2-6.<br><br>**1) Combined motor-cognitive task (dual-task)** requires concurrent performance of balancing on the stability platform and responding to auditory stimuli. Participants will perform 18 trials total in 3 blocks of 6 trials. Each trial will be 30 seconds (s) in duration followed by 30 s of rest (i.e. quiet standing on the platform while holding onto the rails). Between each training block, the participant may step off the platform and rest for 2 minutes.<br>• **Balance task:** requires subjects to stand on a movable platform (Stability Platform, model 16030L, Lafayette Instrument) and to keep the platform in a balanced, horizontal position.<br>• **Auditory reaction time task:** requires subjects to hold two remotes while wearing in-ear headphones and respond to two distinguishable tones that will be played in either the right or left ear across trials of 30 seconds.
**Descriptive measures**	Demographic information, including age, dominant side, gender, ethnicity, race, level of physical activity, height, body weight, co-morbidities, current medications, history of any surgery will be collected on all subjects on visit 1. Performing all these descriptive measures on visit 1 will allow us to determine the inclusion/exclusion of the participant in the study.
**Outcome Measures**	Performance on:<br>1. Auditory reaction time task<br>2. Balance task<br>3. Dual-task (combined auditory reaction time task and balance task)<br><br>Cortical activation:<br>1. Functional near-infrared spectroscopy (fNIRS) assessment<br><br>**Auditory reaction time** will be measured using StimSys reaction time software. Parameters include response accuracy (i.e. appropriate button pressed in response to which ear tone is played) and reaction time in seconds.

	**Performance on the standing balance task** will be quantified by identifying the number of seconds in a 30-second trial that an individual is able to maintain the platform within ±3° of horizontal.<br><br>**Performance on the dual-task** will involve combined auditory reaction time task and balance platform task. Parameters for the auditory reaction time task include response accuracy and reaction time in seconds. Parameters for the balance task include the number of seconds in a 30-second trial that the participant is able to maintain the platform within ±3° of horizontal.<br><br>**fNIRS assessment** will be conducted during baseline and final testing to assess the cortical activity within the prefrontal and sensorimotor areas in response to dual-task training. The NIRS instrument is a non-invasive imaging device that uses low levels of non-ionizing light. It is able to monitor cortical hemodynamics during motor tasks, such as the balance task.<br><br>1. A head cap made of Velcro and plastic materials will be positioned on the participant's head. This cap has several fiber optic cables attached to it. We will need to adjust any hair so that these light sensors can send and receive light from the scalp. This may require us to adjust the position of the cap and fibers to ensure good contact with the skin. The signals from each of the sensors will be verified and adjusted if needed. The setup will take about 15 minutes.
**Order of Experiment/Study Visits**	This study involves 8 total visits. Please refer to Table 1 for a timeline of study visits.<br><br>**Visit 1:**<br><br>1. Trained research personnel will obtain an informed consent.<br>2. If the participant is able to participate in the study and chooses to sign the consent form, we will first record their demographic details such as name, age, date of birth, past medical history, medications, etc.<br>3. After that, we will test their baseline performance of the cognitive task, the motor task, and the dual-task. This will include 6 blocks of 3 trials (18 trials total this day). Each trial will be 30 s in duration, with 30 s of rest following each trial. The following is an example of a testing block, of which order will be randomized: auditory reaction time task, rest 30 s, balance on the dynamic stability platform, rest 30 s, auditory reaction time task while balancing on the platform. Between each block, participants will be given 2 minutes of rest. **Visit 1 will last for approximately 1-1.5 hours.**<br><br>**Visit 2-6:**<br><br>Dual-task training will make up these visits. Participants will perform the balance task and the auditory reaction time task together for 3 blocks of 6 trials (18 trials total). Similar to testing, each training trial will be 30 s in duration followed by 30 s

	of rest. Between training blocks, 2 minutes of rest will be provided. **Visits 2-6 will last for approximately 20-25 minutes each.**<br><br>**Visit 7:**<br><br>Participants will be assessed on their post-training performance of the cognitive task, the motor task, and the dual-task. The testing format will be the same as used in Visit 1 (i.e. 6 blocks of 3 trials), and brain activation will be assessed using the fNIRS. **Visit 7 will last for approximately 30-60 minutes.**<br><br>**Visit 8:**<br><br>1. This visit will occur 1-week after Visit 7. The purpose of this visit is to assess retention effects of the dual-task training intervention.<br>2. The same method will be used to assess retention effects as used on the other testing days (Visits 1 and 7). Participants will perform 6 blocks of 3 trials (18 trials total). **Visit 8 will last for approximately 30-60 minutes.**
**Data Analysis**	Data will be analyzed using a mixed model ANOVA with time (pretest, posttest, and follow-up) as within subject factor.
**Safety Considerations & Monitoring**	The risks of participating in the dual-task training intervention are minimal and are no more than what participants would experience in everyday life.<br><br>**Balance task:** The minimal risk associated with the balance task involves losing balance, but safety measures are in place to ensure participants do not fall. Such safety measures include: a study team member will always be by the side of study participant to guard them; the participant will wear a safety belt around the waist and will be instructed to hold onto the handrails in the event of loss of balance.<br><br>**Auditory task:** The minimal risk associated with the auditory task involves no to minimal discomfort in the ears since the pitch of the auditory stimuli is similar to listening to music or someone over the phone.<br><br>**fNIRS assessment:** There might be some discomfort with the fNIRS cap over the head; however, the discomfort will be minimized by using the appropriately-sized head cap for each study participant.
**Investigators**	PI: Swati Surkar, PhD, PT<br><br>Study Coordinator: Tyler Phinizy, Student, Department of Kinesiology<br><br>Sub-Investigator: Chia-Cheng Lin, PhD, PT
**Number of Centers**	1 center: East Carolina University
**Key References**	Beauchet, O., Dubost, V., Herrmann, F. R., & Kressig, R. W. (2005). Stride-to-stride variability while backward counting among healthy young adults. Journal of neuroengineering and rehabilitation, 2, 26. https://doi.org/10.1186/1743-0003-2-26

	Fraser, S. A., Li, K. Z., Berryman, N., Desjardins-Crépeau, L., Lussier, M., Vadaga, K., Lehr, L., Minh Vu, T. T., Bosquet, L., & Bherer, L. (2017). Does Combined Physical and Cognitive Training Improve Dual-Task Balance and Gait Outcomes in Sedentary Older Adults?. Frontiers in human neuroscience, 10, 688. https://doi.org/10.3389/fnhum.2016.00688<br><br>Ghai, S., Ghai, I., & Effenberg, A. O. (2017). Effects of dual tasks and dual-task training on postural stability: a systematic review and meta-analysis. Clinical interventions in aging, 12, 557–577. https://doi.org/10.2147/CIA.S125201<br><br>Kiss, R., Brueckner, D., & Muehlbauer, T. (2018). Effects of Single Compared to Dual Task Practice on Learning a Dynamic Balance Task in Young Adults. Frontiers in psychology, 9, 311. https://doi.org/10.3389/fpsyg.2018.00311<br><br>Pinti, P., Tachtsidis, I., Hamilton, A., Hirsch, J., Aichelburg, C., Gilbert, S., & Burgess, P. W. (2020). The present and future use of functional near-infrared spectroscopy (fNIRS) for cognitive neuroscience. *Annals of the New York Academy of Sciences*, *1464*(1), 5–29. https://doi.org/10.1111/nyas.13948<br><br>Techayusukcharoen, R., Iida, S., & Aoki, C. (2019). Observing brain function via functional near-infrared spectroscopy during cognitive program training (dual task) in young people. Journal of physical therapy science, 31(7), 550–555. https://doi.org/10.1589/jpts.31.550

Table 1: Order of the 8 study visits. Testing occurs on Study Visits 1, 7, and 8. Dual-task training occurs on Visits 2-6 (consecutive weekdays).

**Assessments**	**Visits**							
	1	2	3	4	5	6	7	8
Consent/<br>Demographic details	✓							
Pre-testing	✓							
Training		✓	✓	✓	✓	✓		

Post-testing							✓	✓

1. Pre-testing includes: fNIRS assessment as well as performance on the- 1) cognitive task, and 2) balance task, and 3) dual-task
2. Training includes: training on the- 1) combined auditory reaction time (cognitive) task and balance (motor) task – i.e. dual-task
3. Post-testing includes: fNIRS assessment as well as performance on the-- 1) cognitive task, and 2) balance task, and 3) dual-task
